# Spatiotemporal Epidemiology of Varicella in Chongqing, China, 2014–2018

**DOI:** 10.3390/ijerph17020662

**Published:** 2020-01-20

**Authors:** Hua Zhu, Han Zhao, Rong Ou, Qing Zeng, Ling Hu, Hongfang Qiu, Manoj Sharma, Mengliang Ye

**Affiliations:** 1 Department of Epidemiology and Health Statistics, School of Public Health and Management, Chongqing Medical University, Chongqing 400016, China; 2018110993@stu.cqmu.edu.cn (H.Z.); kingzeng1@126.com (Q.Z.); 2018111007@stu.cqmu.edu.cn (L.H.); qhf2644398937@163.com (H.Q.); 2 Chongqing Municipal Center for Disease Control and Prevention, Chongqing 400042, China; molly_sunny2012@163.com; 3 Department of Medical Informatics Library, Chongqing Medical University, Chongqing 400016, China; ourong@cqmu.edu.cn; 4 Department of Behavioral and Environmental Health, Jackson State University, Jackson, MS 39213, USA; manoj.sharma@jsums.edu

**Keywords:** varicella, epidemiology, spatial analysis, spatiotemporal cluster, Chongqing

## Abstract

Although immunization against varicella using vaccines has been proven to be significant and effective in the past decades, varicella remains a major public health concern for many developing countries. Varicella vaccination has not been introduced into routine immunization programs in China, and varicella outbreaks have continued to occur. Taking the city of Chongqing, which has a high prevalence of varicella, as an example, this study explored the spatiotemporal epidemiology of varicella. Based on the reported data of varicella cases from 1 January 2014 to 31 December 2018 in Chongqing, hot spots and space-time clusters of varicella were identified using spatial autocorrelation analysis and scan statistics. Within this period, a total of 112,273 varicella cases were reported in Chongqing (average annual incidence: 73.44 per 100,000), including one death. The incidence of varicella showed an increasing trend with significant seasonal peaks, which occurred during April to July and October to January of the following year. The total ratio of male to female patients affected was 1.10:1. Children under the age of 15 and students accounted for the majority of the patient population. The hotspots detected through local spatial autocorrelation analysis, and the most likely clusters identified by scan analysis, were primarily in the main urban districts of Chongqing. The secondary clusters were mostly detected in northeast and southwest Chongqing. There were obvious spatial dependence and spatiotemporal clustering characteristics of varicella in Chongqing from 2014 to 2018. High-risk districts, populations, and peak periods were found in this study, which could be helpful in implementing varicella prevention and control programs, and in adjusting vaccination strategies for the varicella vaccine based on actual conditions.

## 1. Introduction

Varicella is an acute and highly contagious respiratory infection caused by the varicella-zoster virus, and it predominantly affects children [1,2]. Varicella is characterized by a generalized vesicular exanthema, usually accompanied by fever and malaise [3]. In addition, this disease can lead to serious complications, such as secondary bacterial infections and central nervous system involvement, resulting in hospitalizations and deaths [4,5]. Following primary infection, the virus remains latent in nerve cells and may be reactivated later to cause the more serious secondary infection, herpes zoster (shingles) [6,7,8]. Varicella can be prevented by active immunization, and the varicella vaccine has now been licensed and used as a monovalent or combined with the measles, mumps, and rubella vaccines [9]. In some countries with high immunization coverage, such as America, Uruguay, Canada, and Australia, a marked decline in the morbidity of varicella and varicella-related hospitalization and complication rates has been reported since the introduction of varicella vaccines into routine immunization programs [10,11,12,13,14,15]. In contrast, the varicella vaccine in China is a category II vaccine, which is not free. Residents can choose whether they want to be inoculated or not, and it is not included in the national expanded immunization program for those with limited economic capabilities. Unlike the national infectious diseases listed by the Ministry of Health, varicella has not been managed and monitored in a systematic manner, and only public health emergencies related to varicella have been reported. In China, the annual average incidence of varicella from 2005 to 2015 increased by 30%, and the number of varicella patients is still on the rise [16]. The damage and economic burden caused by varicella are becoming increasingly severe, hence bringing the epidemic situation under control is a serious problem being considered in China. 

The World Health Organization (WHO) has suggested that an appropriate disease surveillance system should be established to assess the disease burden of varicella before introducing routine childhood varicella immunization, and sustained monitoring should be carried out after the vaccine is included in the immunization program [9]. Therefore, effective monitoring of the varicella epidemic situation is helpful to understand the epidemiological characteristics and epidemic trends of varicella, and further provides the basis for the formulation of prevention and control strategies in the future. Given the high prevalence and rising trend of varicella incidence in the city of Chongqing, the Health and Family Planning Commission has included varicella as a notifiable Class C communicable disease (an infectious disease that requires proper surveillance) since 2014 [17]. This action requires that the infectious disease report cards of all patients with varicella must be filled out by their doctors after diagnosis, and reported to the internet or sent to health institutions responsible for reporting within 24 h [17,18]. Spatiotemporal epidemiological approaches based on geographic information systems (GIS) are widely used to understand the geographical distribution and space-time characteristics of various infectious diseases [19,20,21]. However, previous studies on the spatial heterogeneity and spatial-temporal features of varicella have been limited to a few areas, including Valencia City in Spain [22], the Republic of Korea [23], and Guangxi Province in China [24]. Chongqing, situated in the southwest of China and upriver of the Yangzi River, is the only interior municipality in the central and western regions of China with a large area and population. Moreover, to the best of our knowledge, no researcher has explored the spatiotemporal patterns of varicella in Chongqing.

The purpose of our study was to describe the epidemiological characteristics of varicella in Chongqing in 2014–2018, and investigate the spatial variations and spatiotemporal features of varicella at the county level. The results could provide useful information for health administrative departments to formulate preventive and control policies, as well as vaccination promotion strategies.

## 2. Materials and Methods 

### 2.1. Data Source and Case Definition

The data of all varicella cases between 2014 and 2018 were gathered from the communicable disease surveillance network system of Chongqing Municipal Center for Disease Control and Prevention, including the sex, age, occupation, diagnosis date, death date, and residential addresses of patients with varicella. Notably, these obtained data did not include patients with herpes zoster. The demographic data of 38 counties and districts were derived from the Statistical Yearbooks of Chongqing. 

A suspected case of varicella is defined as a patient with pruritic papule and vesicular rash on the skin and mucous membranes, with or without fever and headache, and without the possibility of other eruptive diseases. Confirmed cases are clinically diagnosed cases and laboratory-confirmed cases. The former refers to suspected cases during the varicella epidemic season or in those with a history of contact with varicella or herpes zoster patients in the 2–3 weeks preceding the onset of illness. The latter refers to a suspected case accompanied by any of the following items: the immunoglobulin M (IgM) antibody of the varicella-zoster virus was positive without varicella vaccine inoculation in the last month, varicella zoster virus was isolated, or the antigen of the varicella-zoster virus was detected by direct fluorescence assay (DFA) or polymerase chain reaction (PCR), or the titer of the immunoglobulin G (IgG) antibody of the varicella-zoster virus alongside the sera increased more than four-fold at 2–4 week intervals.

### 2.2. Study Area

Chongqing, the largest municipality under the direct control of the national government, is located in southwest China between longitudes 28°10′ N–32°13′ N and latitudes 105°11′ E–110°11′ E. The city governs 38 districts and counties, with a total area of 824,000 square kilometers and a population of 31.02 million. It is a mountainous city and has a subtropical humid monsoon climate characterized by hot and rainy summers, and warm and wet winters. The main urban area of Chongqing covers nine districts (Yuzhong, Jiangbei, Nan’an, Jiulongpo, Shapingba, Dadukou, Beibei, Yubei, and Banan) and is the political, economic, and cultural center of the city and metropolitan area, with high level of economic development. 

### 2.3. Statistical Analysis

#### 2.3.1. Descriptive and Geographical Analysis

The epidemiological situation and characteristics (i.e., age, gender and occupation distribution, time trend, and seasonal pattern) of varicella cases from 2014 to 2018 in Chongqing were described. The annual incidence of varicella per 100,000 persons in each district and county was calculated and inputted into the map of Chongqing. In the annual incidence maps of varicella, the incidence rates in different regions were divided into nine levels and represented by different colors. Excess hazard ratios were computed and visualized in excess hazard maps, using Geoda software to display the risk of varicella in each county relative to the average level. The excessive risk ratio refers to the ratio of actual incidence of a certain county over the average incidence of the whole area [25].

#### 2.3.2. Spatial Autocorrelation Analysis

Spatial autocorrelation refers to the spatial dependence between nearby spatial units of the same variable, and shows the correlation within variables across space [26]. We performed global and local autocorrelation analysis using Geoda software to investigate the spatial autocorrelation of the morbidity of varicella in Chongqing. The former was used to explore spatial dependence in the whole area, while the latter was adopted to further determine the patterns and locations of the local counties with spatial autocorrelation. The global Moran’s I varied from −1 to 1 as the indicator of global spatial autocorrelation, and the inference for Moran’s I statistic was based on a null hypothesis of spatial randomness. A positive or negative value implied a positive or negative spatial autocorrelation, respectively. In the local indicators of the spatial autocorrelation cluster map, the spatial association pattern among local counties was classified into four categories [27]: high-high clusters, low-low clusters, low-high outliers, and high-low outliers. Notably, the high-high clusters are the important “hot spots” for varicella transmission and occurrence.

#### 2.3.3. Spatial and Space-Time Clustering Analysis

We performed purely spatial and space-time scan analyses to discover clusters with high rates at the district/county level on the basis of the discrete Poisson probability model, using SaTScan software. Spatial and spatio-temporal scan statistics are defined by a circular window corresponding to the geographic areas on the map and a cylindrical window with a circular geographic base and with the height corresponding to time, respectively [28]. The scanning window is moved in space and/or time to obtain an infinite number of overlapping windows with different sizes and shapes, jointly covering the whole study area. Each circle or cylinder is a possible candidate cluster. The maximum geographical circle size was set as 25% of the total population, and the maximum temporal scan period was set as 50% of the whole study period. Geographically overlapping clusters were not reported by default. For each scanning window, the null hypothesis was that the risks were the same inside and outside the window, whereas the alternative hypothesis was that there is an elevated risk within the window. The log likelihood ratio (LLR) was used as the test statistic, and the *p*-value was obtained through Monte Carlo hypothesis testing with 999 replications. The window with the largest LLR was viewed as the most likely cluster, and the other windows with statistically significant LLRs were the secondary clusters, which were ranked in order of their LLR values. Relative risk, a ratio of the estimated risk within the window to outside the window, was also used to estimate the risk of the cluster.

#### 2.3.4. Statistical Software

The arrangement and management of varicella surveillance data were performed in Excel 2016 (Microsoft, Redmond, WA, USA). The software used for spatial analysis included Geoda version 1.12 and SaTScan™ version 9.5 (Martin Kulldor, National Cancer Institute, Bethesda, MD, USA). ArcGIS software 10.2.2 (ESRI Inc., Redlands, CA, USA) was used for the mapping. An χ^2^ test was conducted using SPSS version 22.0 (SPSS Inc., Chicago, IL, USA). *p* < 0.05 with two sides was considered statistically significant for all the tests.

## 3. Results

### 3.1. Epidemiological Characteristics

**Figure 1:**
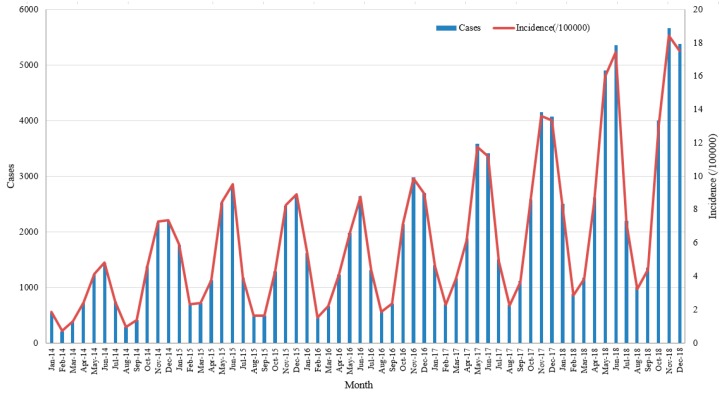
Monthly distribution of varicella in Chongqing from 2014 to 2018.

There were 112,273 varicella cases and one fatal case reported in Chongqing City between 2014 and 2018, with an average annual incidence of 73.44 per 100,000, including 559,033 male cases and 336,396 female cases. The prevalence of varicella showed a clear upward trend during the five-year study period, and the annual incidence rate increased from 39.06 per 100,000 in 2014 to 119.46 per 100,000 in 2018. The average male morbidity (67.64 per 100,000) was higher than female morbidity (64.89 per 100,000) (χ^2^ = 47.99, *p* < 0.001), with a male-to-female ratio among all cases of 1.10:1. With respect to the age distribution, the overwhelming majority of cases were in patients under 15 years old, at 80.0% of the total. In all occupation groups, students accounted for the largest proportion (60.7%) of cases, followed by kindergarten children (18.8%) and scattered children (9.5%). The demographic characteristics of varicella cases from 2014 to 2018 are shown in Table 1. The monthly distribution of varicella cases in Chongqing presented a clear seasonal variation, and two significant incidence peaks in April to July and October to January of the following year (Figure 1). Moreover, the number of cases in the second peak (47.4%) was generally larger than that in the first peak (40.7%).

### 3.2. Spatial Distribution

**Figure 2:**
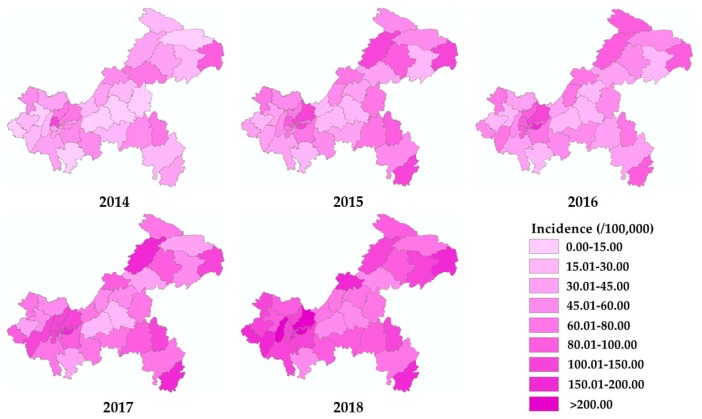
Incidence maps of varicella in Chongqing from 2014 to 2018.

**Figure 3:**
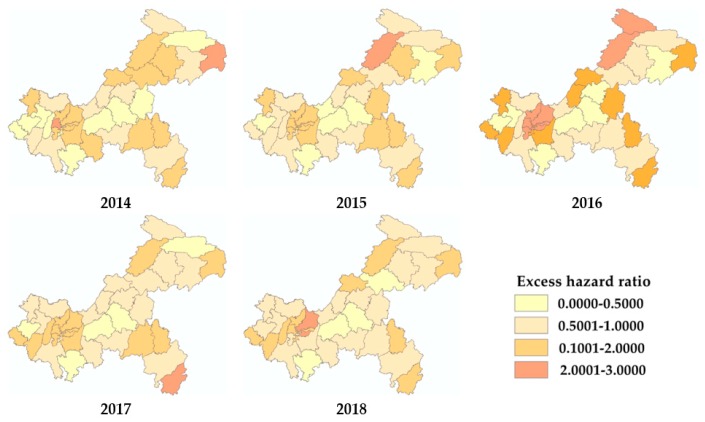
Excess hazard maps of varicella in Chongqing from 2014 to 2018.

The geographical distribution of varicella incidence at the district level in Chongqing from 2014 to 2018 is shown in Figure 2. The incidence increased in almost all districts and counties. The number of districts with an incidence over 100 increased from 1 in 2014 to 21 in 2018. The top five counties with the highest average incidence over five years were Yubei, Nanan, Wushan, Xiushan, and Kaixian. Figure 3 illustrates the excess hazard ratio of varicella in Chongqing.

### 3.3. Spatial Autocorrelation Analysis 

**Figure 4:**
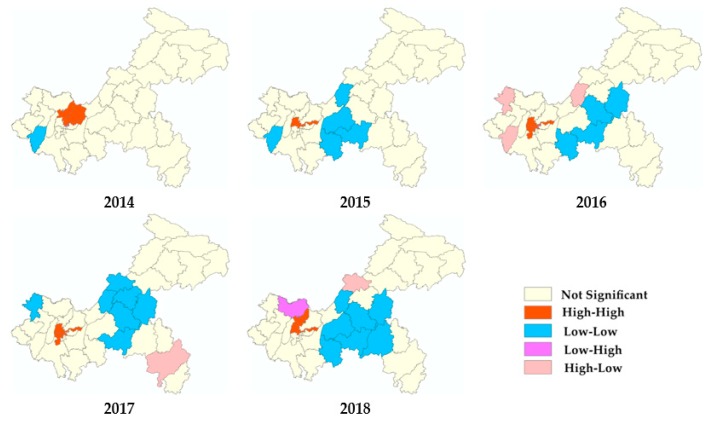
Local spatial autocorrelation cluster maps of varicella in Chongqing in 2014–2018.

Global spatial autocorrelation showed that all the values of global Moran’s I were positive, but only the value in 2016 was statistically significant (Table 2). Consequently, a positive spatial autocorrelation existed in 2016, and the spatial distribution of varicella was random as a whole in other years. Statistically significant local spatial clusters were identified by local spatial autocorrelation analysis at the county level in Chongqing (Figure 4). The locations and regions of the four spatial clusters were not exactly the same every year. Notably, the districts of all the hot spots were detected in the main urban areas of Chongqing, including Beibei, Yubei, Jiangbei, Shapingba, and Jiulongpo.

### 3.4. Cluster Analysis 

**Figure 5:**
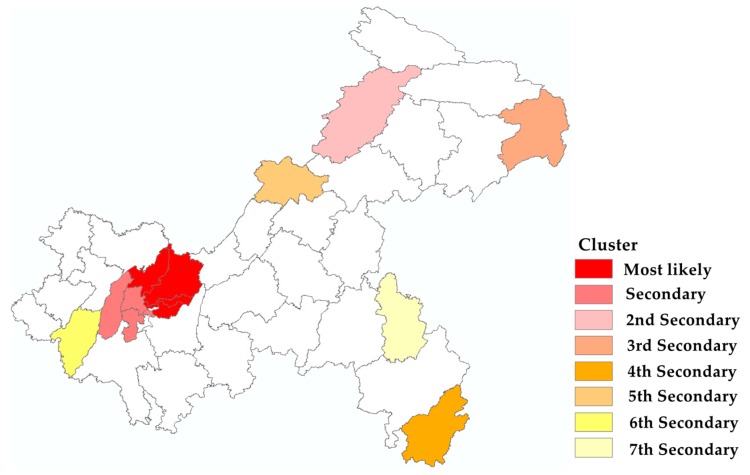
Spatial cluster map of varicella in Chongqing, 2014 to 2018.

From 2014–2018, there were eight statistically significant space clusters for a high incidence of varicella detected purely using a space scan, including one most likely cluster and seven secondary clusters (Figure 5 and Table 3). The most likely clusters covering four districts (Nanan, Jiangbei, Beibei, and Yubei) and the secondary cluster covering three districts (Shapingba, Jiulongpo, and Bishan) were mainly located in the main urban areas. The second secondary cluster to the seventh secondary cluster were detected in the districts (counties) of Kaixian, Wushan, Xiushan, Liangping, Yongchuan, and Qianjiang. 

**Figure 6:**
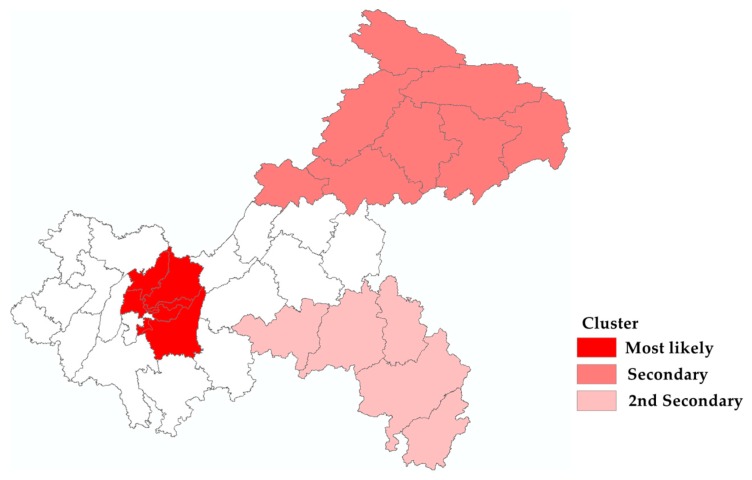
Spatial-temporal cluster map of varicella in Chongqing, 2014 to 2018.

Three statistically significant spatiotemporal clusters were identified by the space-time scan (Figure 6 and Table 4). The most likely cluster occurred between October 2016 and December 2018, including eight of the nine main urban districts of Chongqing. The secondary cluster (*n* = 8) and the second secondary cluster (*n* = 5) were mainly in the northeast from October 2018 to December 2018 and in the southwest of Chongqing from November 2017 to December 2017.

## 4. Discussion

Our research determined the basic epidemiological features of varicella in Chongqing, and confirmed spatial and spatial-temporal clusters of varicella using the spatial analysis technologies of GIS, which will be helpful for health institutions to control varicella and reasonably carry out public health planning and resource allocation. The incidence of varicella in Chongqing dramatically increased to a high level from 2014 to 2018, and the overall prevalence in Chongqing was higher than the values previously reported [29] and the rates nationwide or in some other provinces during the same period [16,30,31,32], which indicated that varicella is a growing and serious public health problem. A rising trend was also observed in the whole country and in other Chinese provinces [16,30,31]. This may be the result of the low vaccination coverage rate of varicella, low serum antibody concentration, accumulation of a susceptible population, and the continuous occurrence of breakthrough cases [33,34]. Vaccination with live attenuated varicella is a significant and effective way to control the varicella epidemic and offer protection against varicella [35,36,37]. Due to the principle of voluntary choice and self-funded payments, and the neglected risk of varicella compared to other infectious diseases such as measles and mumps, the vaccination rate in many parts of China is relatively low and does not reach the 80% level that the World Health Organization recommends [32,33,38,39]. Therefore, the immunity barrier is insufficient for susceptible persons, especially in relatively underdeveloped regions. To standardize varicella vaccination and prevent continued outbreaks and high prevalence of varicella, the Chongqing Health and Family Planning Commission issued a guidance on varicella vaccination, and recommended the use of two doses of varicella vaccine in immunization schedules in October 2018 [40]. We assume that this official recommendation will have a preventive and control effect on the epidemic of varicella, but we cannot infer the subsequent epidemic situation from our current data. The specific immune coverage and sero-epidemiological characteristics of varicella among the people in Chongqing are not clear, and are worth studying in the future.

Consistent with previous studies [16,29,41], the number of male cases is always higher than the number of female cases, which may be due to males participating in more activities, having worse health habits, and having more opportunities to be infected. The incidence of varicella in Chongqing has significant seasonal variation and peaks, which are approximately coincident with findings from studies in other areas [30,31,32]. Moreover, several studies [42,43,44] have suggested that the variations in incidence peak in different regions may be influenced by different temperatures, ultraviolet (UV) light, geographical locations, population density, climate models, and other potential factors [45,46,47,48]. Densely populated learning environments and relatively concentrated activity spaces where students and kindergarten children gather undoubtedly increase the incidence and spread of viruses in these groups, which make them the main population for varicella. Additionally, the majority of patients were children and teenagers under 15 years old, consistent with results found in previous studies [16,31,41]. Accordingly, education and health administrative departments should place importance on improving the awareness of students, parents, and teachers around infectious diseases and vaccination, and strengthening the monitoring of epidemic situations in schools and kindergartens.

In the present study, the spatial analysis implied that the morbidity of varicella in Chongqing was not randomly distributed in space, although the overall spatial autocorrelation of this disease was not strong. The high-high clustering districts detected by local spatial autocorrelation all belonged to the main urban area of Chongqing. The incidence map, excess hazard map, and scan statistics also revealed that the main urban region was the hot spot for varicella. Several features in the main urban area, including high population density, a developed economy, and frequent population migration, might be the reasons for the epidemic of infectious diseases here [49]. Pure spatial and spatiotemporal scans also revealed secondary clusters of varicella in some districts and counties of the northeast and southwest Chongqing, areas with relatively backward economies. The poor regional level, accompanied by the low average economic income of families and poor awareness of vaccines or disease-related knowledge, hinders the distribution of voluntarily self-paid vaccines [50,51,52]. Reducing the cost of the varicella vaccine, giving relevant vaccination subsidies, or including varicella vaccines in the reimbursement scope of basic medical insurance are suggested to improve immunization coverage. A number of studies and analyses have indicated that two doses of varicella vaccine are highly effective, and produce stronger protection than one dose [36,53,54]. Considering the current high prevalence of varicella, health institutions should consider the inclusion of varicella vaccines in the national immunization program, and should also highly recommend a second dose of vaccine to better control the spread of varicella, particularly in hyper endemic areas. The locations of high-risk areas determined by various spatial analyses in the present study are slightly different to those found in previous studies, which may be caused by different principles and incorporated variables. However, there is an overall consistency that suggests the robustness of the results.

Despite the findings above, several limitations of our study should be acknowledged. First, our data were obtained from the official disease-monitoring system, and we cannot rule out the possibility of under- or over-reporting for various reasons [55,56,57], such as failure to seek treatment among patients with mild symptoms, and inadequate diagnosis among clinically diagnosed cases. However, the proportion of these people is likely to be small and stable [58,59,60], and thus will not affect the epidemic characteristics and transmission trends of the disease. Second, we obtained only five-year reporting data during a relatively short period, which was not sufficient to develop understanding of the long-term epidemic characteristics and periodical trend changes. Therefore, with the development of disease surveillance, more data should be collected and analyzed in the future. Finally, in view of insufficient information on geographic, climatic, socio-economic, and other possible risk factors, we have not conducted a more in-depth study to assess their relationships and relevance, so it is difficult to determine the reasons for the clusters.

## 5. Conclusions

The varicella incidence in Chongqing has increased since 2014 to a high level in 2018, with evident seasonal variation, and several spatial and spatiotemporal high-risk clusters of varicella exist. These clusters were mainly located in the main urban areas and the northeastern and southwestern regions of Chongqing. Children under the age of 15 and students were the predominant varicella patients. To better control this epidemic and reduce the burden of this disease, continuous monitoring and management of varicella is necessary, especially in those endemic areas, and health authorities should widely disseminate disease knowledge among the people and develop new immunization strategies to increase the inoculation rate and the overall immunity level.

## Figures and Tables

**Table 1:** Demographic characteristics of varicella cases in Chongqing, 2014–2018.

	2014	2015	2016	2017	2018	Total
	N (%)	N (%)	N (%)	N (%)	N (%)	N (%)
**Age (year)**						
0~	323 (2.8)	597 (3.3)	607 (3.2)	837 (3.2)	932 (2.5)	3296 (2.9)
1~	383 (3.3)	535 (2.9)	652 (3.4)	786 (3.0)	975 (2.6)	3331 (3.0)
2~	246 (2.1)	388 (2.1)	410 (2.2)	544 (2.1)	769 (2.1)	2357 (2.1)
3~	429 (3.7)	681 (3.7)	653 (3.4)	886 (3.4)	1531 (4.1)	4180 (3.7)
4~	660 (5.7)	1039 (5.7)	988 (5.2)	1247 (4.8)	1888 (5.1)	5822 (5.2)
5~	995 (8.5)	1440 (7.9)	1223 (6.4)	1751 (6.7)	2452 (6.6)	7861 (7.0)
6~	1124 (9.6)	1710 (9.4)	1494 (7.9)	1866 (7.1)	2841 (7.6)	9035 (8.1)
7~	1204 (10.3)	1830 (10.0)	1770 (9.3)	2245 (8.6)	3031 (8.2)	10,080 (9.0)
8~	947 (8.1)	1686 (9.2)	1548 (8.1)	2265 (8.6)	3102 (8.4)	9548 (8.5)
9~	746 (6.4)	1192 (6.5)	1240 (6.5)	1965 (7.5)	2622 (7.1)	7765 (6.9)
10~	2188 (18.7)	3802 (20.8)	4283 (22.5)	6406 (24.4)	9764 (26.3)	26,443 (23.6)
15~	1095 (9.4)	1438 (7.9)	1878 (9.9)	2406 (9.1)	3211 (8.7)	10,028 (8.9)
20~	565 (4.8)	715 (3.9)	785 (4.1)	1023 (3.9)	1216 (3.3)	4304 (3.8)
25~	456 (3.9)	729 (4.0)	841 (4.4)	980 (3.7)	1211 (3.3)	4217 (3.8)
30~	224 (1.9)	331 (1.8)	449 (2.4)	708 (2.7)	1014 (2.7)	2726 (2.4)
35~	99 (0.8)	156 (0.9)	201 (1.1)	328 (1.2)	496 (1.3)	1280 (1.1)
**Gender**						
Male	6172 (52.8)	9709 (53.1)	10,062 (52.9)	13,742 (52.4)	19,212 (51.8)	58,897 (52.5)
Female	5512 (47.2)	8560 (46.9)	8960 (47.1)	12,501 (47.6)	17,843 (48.2)	53,376 (47.5)
Sex ratio	1.12	1.13	1.12	1.10	1.08	1.10
**Occupation**						
Scattered children ^1^	1226 (10.5)	1901 (10.4)	1963 (10.4)	2455 (9.4)	3108 (8.4)	10,653 (9.5)
Kindergarten children	2397 (20.5)	3842 (21.0)	3406 (17.9)	4562 (17.4)	6946 (18.7)	21,153 (18.8)
Student	6729 (57.6)	10,613 (58.0)	11,400 (59.9)	16,309 (62.1)	23,149 (62.5)	68,200 (60.8)
others	1332 (11.4)	1913 (10.47)	2253 (11.8)	2917 (11.1)	3852 (10.4)	12,267 (10.9)
Total	11,684	18,269	19,022	26,243	37,055	112,273

Note: ^1^ Scattered children refers to children who have not yet reached the age of 3 years (the age for attending kindergarten), or who are taken care of by their family members.

**Table 2:** Global spatial autocorrelation of varicella in Chongqing, 2014–2018.

Year	Moran’s I	Z-Score	*p*-Value
2014	0.0274	0.5354	0.272
2015	0.1049	1.1856	0.124
2016	0.2030	2.2173	0.021
2017	0.0383	0.6808	0.249
2018	0.1048	1.2736	0.109

**Table 3:** Results for spatial clusters of varicella in Chongqing from 2014 to 2018.

Cluster Type	Counties (*n*)	Observed/Expected	Radius (km)	Relative Risk	Log Likelihood Ratio	*p*-Value
Most likely	4	1.68	31.61	1.89	3517.23	<0.001
Secondary	3	1.32	22.23	1.36	577.31	<0.001
2nd Secondary	1	1.52	0	1.55	518.26	<0.001
3rd Secondary	1	1.60	0	1.62	263.35	<0.001
4th Secondary	1	1.54	0	1.55	227.48	<0.001
5th Secondary	1	1.21	0	1.22	51.92	<0.001
6th Secondary	1	1.15	0	1.16	48.63	<0.001
7th Secondary	1	1.20	0	1.21	34.17	<0.001

**Table 4:** Results for space-time clusters of varicella in Chongqing from 2014 to 2018.

Cluster Type	Cluster Time	Counties (*n*)	Observed/Expected	Radius (km)	Relative Risk	Log Likelihood Ratio	*p*-Value
Most likely	2016/10–2018/12	8	1.98	31.18	2.25	5379.65	<0.001
Secondary	2018/10–2018/12	8	2.66	166.76	2.71	1098.91	<0.001
2nd Secondary	2017/11–2017/12	5	3.95	160.56	3.98	717.76	<0.001
